# A New Self-Calibrated Procedure for Impact Detection and Location on Flat Surfaces

**DOI:** 10.3390/s130607104

**Published:** 2013-05-30

**Authors:** José A. Somolinos, Amable López, Rafael Morales, Carlos Morón

**Affiliations:** 1 GITERM. ETS I. Navales, Universidad Politécnica de Madrid, Arco de la Victoria 4, Madrid 28040, Spain; E-Mail: amable.lopez@upm.es; 2 E.T.S.I. Industriales, Universidad de Castilla-La Mancha, Albacete 02071, Spain; E-Mail: Rafael.Morales@uclm.es; 3 Sensors and Actuators Group, Juan de Herrera 6, Madrid 28040, Spain; E-Mail: carlos.moron@upm.es

**Keywords:** impact detection and location, acoustic wave propagation, robotic based experimental setup

## Abstract

Many analyses of acoustic signals processing have been proposed for different applications over the last few years. When considering a bar-based structure, if the material through which the sound waves propagate is considered to be acoustically homogeneous and the sound speed is well known, then it is possible to determine the position and time of impact by a simple observation of the arrival times of the signals of all the transducers that are strategically disposed on the structure. This paper presents a generalized method for impact detection and location on a flat plate, together with a calibration procedure with which to obtain the sound speed from only one set of measurements. This propagation speed is not well known as a result of either imprecise material properties or the overlapping of longitudinal and transversal waves with different propagation velocities. The use of only three piezoelectric sensors allows the position and time of impact on the flat plate to be obtained when the sound speed is well known, while the use of additional sensors permits a larger detection area to be covered, helps to estimate the sound speed and/or avoids the wrong timing of difference measurements. Experimental results are presented using a robot with a specially designed knocking tool that produces impacts on a metallic flat plate.

## 1. Introduction

Many analyses of acoustic signals measurement and processing have been carried out over the last few years for different and diverse purposes. Examples of experimental applications such as damage detection, failure prevention or interactive human-machine interfaces [1–3], among others, have been studied using these techniques. The use of *Time of Arriving* (TOA) measurements is a common technique in various application fields such as ultrasonic sensorial systems in the food industry [4,5] or the detection of fishing banks and the depth of the seabed in assisted navigation tools.

In other applications, when the estimation of impact forces on a structure or on a machine is required, the knowledge of the position in and time instant at which the impact was produced must be determined beforehand. Various methods have been proposed in literature. For example, Martin and Doyle [6] identified impact forces by using deconvolution analysis from acceleration measurements, and Nu *et al.* [7] used lead-zirconate-titanate (PZT) piezoelectric sensors for strain measurements and impact force estimations.

The problem of time delay estimation of acoustic signals has been solved by using correlation methods [8] or other complex algorithms [9] to identify the first time arrival time in the presence of several modes, reflections or other wave distortions. Using the simple technique of measuring TOA, the method proposed in [10] considers the differences in the propagation time of an acoustic wave in a metallic (homogeneous, acoustically isotropic material)-based structure, and it determines the time instant and the position at which the impact or collision is produced (details in [10–12]). This procedure is not focused on the detection of structural damages or force estimations, but is focused solely on the detection and location of the position and time instant of the impact. A simple, robust and low cost electronic device was therefore designed (details in [13,14]) to measure differences in propagation times. It is based on a three-stage circuit topology for each pair of piezoelectric sensors. The first stage is a double high impedance linear amplifier in which two independent voltage signals from a pair of PZT wafer piezoelectric sensors [15] are connected. After a precision signal full rectifier stage, an edge signal detector produces a digital rising step signal from each of the amplified signals. From any set of pairs of step signals (up to five pairs) digital counter time differences (with a base clock signal of up to 16 MHz from the computer bus to which the circuit is connected) are obtained. This last digital opto-isolated digital circuit stage was developed under the M-module [16] and the VITA open standards [17], and can be integrated into any computer provided with a VME, VXI, ISA, or PCI bus with its corresponding carrier board, together with its custom made low level driver (developed for both ISA and VME buses [14]).

However, if impacts are produced on a thin metallic plate (an acoustically isotropic material), the speed of propagation of the waves is not well determined because of the coupling of the longitudinal and transversal waves with different speeds during their propagation [18], and this speed must be determined beforehand in order to achieve an accurate impact position. What is more, if the plate is not flat, *i.e.*, it has holes (see [19]) or is made of a non-isotropic material [20], more complex algorithms are needed to locate the impact point from the piezoelectric sensors, in addition to different techniques such as triangulation, lamb waves or wavelet transforms, among others [21–23].

This paper presents a very simple algorithm with which to detect and locate an impact on a thin metallic plate based on simple differences of TOA, even when the speed of propagation is not well determined. It is based on triangulation techniques and consists of the evaluation of all the possible intersections between hyperbola branches with signs and the computation of the sum of the squared distances from which average speed of propagation can be obtained as an optimization algorithm with not too many iterations.

The paper is organized as follows: Section 2 details the algorithm for impact detection and location on a flat plate when the sound speed is known, and the algorithm for estimation of the speed of propagation is also presented. In Section 3, an experimental setup based on a robotized system is briefly described, and some experimental results are illustrated in Section 4 in order to validate the effectiveness of the proposed method. Finally, in Section 5, some conclusions and suggestions for further work are briefly outlined.

## 2. Impact Detection and Location

A flat plate of a homogeneous material as regards its acoustic properties (e.g., metallic) is considered. The flat plate is also considered to be isotropic. Piezoelectric sensors are located at known positions. Without any loss of generality, a sensor denoted as 1 is placed at the origin of a 2D Cartesian frame. The point at which the impact is produced is defined as P_Im_ = (x_Im_ y_Im_)^T^ and the position of the j-th sensor with regard to the absolute location of the i-th sensor P_i_, i = 1, 2, … n − 1 is denoted as P_ij_ = (x_ij_ y_ij_)^T^ for i = 1, 2, … n − 1, i < j ≤ n, where P_ii_ = (x_ii_ y_ii_)^T^ = (0 0)^T^. If the constant sound velocity C is known, then the propagation time of the fundamental acoustic wave from P_Im_ to each of the n sensors is computed as:

(1) $$t_{i}=\frac{\left\|P_{i\;\text{Im}}\right\|}{C}\text{for}\;i=1,2,\cdots ,n$$

where ‖*P_i Im_*‖ is the distance between the impact point P_Im_ and the position of the i-th sensor.

These times cannot be directly measured because the instant at which the impact was produced is not known. An analog electronic conditioner located near the piezoelectric sensors that was developed for up to 10 piezoelectric sensors allows the acoustic wave received by each of the piezoelectric sensors to be converted into a rising edge voltage signal. This first stage of each of the individual conditioners is responsible for impedance adaptation, current amplification, adjustable level detection and the differential transmissions of edge signals.

After this initial analog processing and wire transmission, an opto-isolation intermediate stage provides electric isolation and conversion into TTL digital signals with constant and deterministic delays. Differences in times of arrival (TOA) can then be obtained by simple time counting using digital counters with a high frequency clock signal. The digital stage of the conditioner was developed under the M-module standard [16] and the VITA open standards [17], and can be used with different carrier boards for diverse domestic or instrumentation buses (such as PCI, CPCI, VXI).

**Figure 1.**
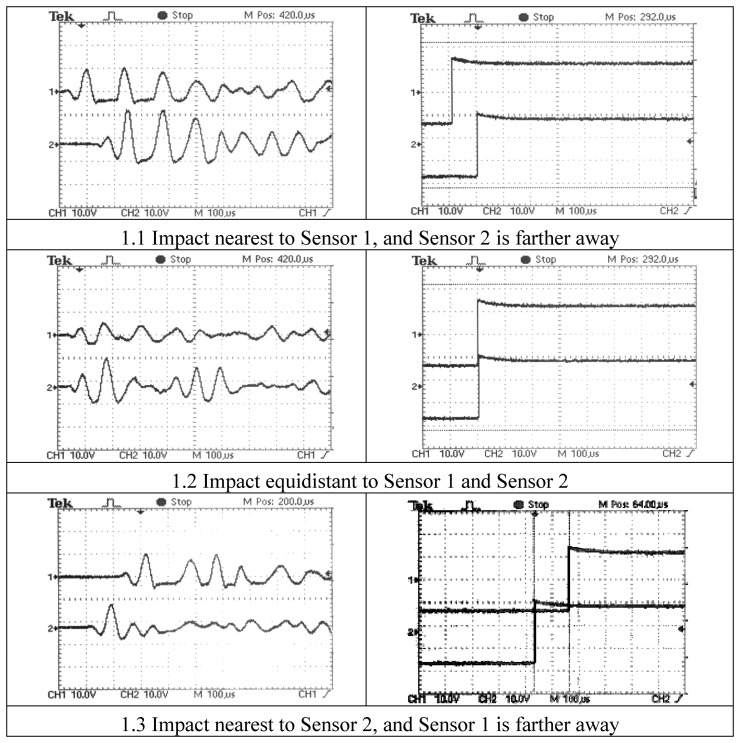
(**a**) Left. Amplified signals from sensors; (**b**) Right. Edge signals from the analog stage of the conditioner.

Figure 1(a) shows three pairs of amplified signals from two piezoelectric sensors (Sensor 1 and Sensor 2) on a metallic plate, while Figure 1(b) depicts the corresponding three pairs of rising edge signals from the opto-isolator inputs before their conversion into 0–5 V signals for digital counting.

### 2.1. When Sound Propagation Speed is Well Known

After computing the time differences, any of the sensors may be used as a reference sensor. From any pair of sensors i and j, the time differences are signed values according to:

(2) $$\begin{array}{ll} \Delta t_{ij}=t_{i}-t_{j}=\frac{\left\|P_{\text{i Im}}\right\|}{C}-\frac{\left\|P_{\text{j Im}}\right\|}{C} & \text{for}\;i=1,2,\cdots ,n\;\text{and}\;i<j\leq n \end{array}$$

where ‖ ‖ denotes Cartesian norm and C, denotes the sound speed propagation which is considered to be well known. These time increments are signed times according to:

(3) $$\Delta t_{ij}=\{\begin{array}{ccc} >0 & if & \left\|P_{\text{i Im}}\|>\|P_{\text{j Im}}\right\|\\ =0 & if & \left\|P_{\text{i Im}}\|=\|P_{\text{j Im}}\right\|\\ <0 & if & \left\|P_{\text{i Im}}\|<\|P_{\text{j Im}}\right\| \end{array}$$

In the most general form, when an impact is detected by any of the sensors, a set of n_H_ differences of time is obtained, where n_H_ is:

(4) $$n_{H}=\left(\begin{array}{c} n\\ 2 \end{array}\right)=\frac{n!}{2!\cdot (n-2)!}=\frac{n\cdot (n-1)}{2}$$

These time differences can be converted into distance differences by using the following expression:

(5) $$C\cdot \Delta t_{ij}=\|P_{\text{i Im}}\|-\|P_{\text{j Im}}\|\text{for}\;i=1,2,\cdots ,n-1,i<j\leq n$$

According to Equation (3), these distances given by Equation (5) are also signed distances. In other words, if the distance between the impact position and the i-th sensor is smaller than the distance between the impact position and the j-th sensor, then the distance computed from Equation (5) will be negative, equal to zero if both distances are identical, or positive in the other case.

A hyperbola, meanwhile, is a conic curve that can be defined as the locus of points where the differences of the distances to the two points called foci is a constant 2. a, where a retains the geometrical meaning of the semi-major axis. The hyperbola is thus composed of two branches, one of which corresponds with the positive constant differences and the other of which corresponds with the negative constant differences.

After computing these time differences, they can be grouped and sorted from lowest to highest absolute value in order to process the information with regard to the sensor that is nearest to the impact, as occurred in [11]. Only n − 1 time differences are therefore independent measurements, and the algorithm used to determine the impact position can be applied from only these n − 1 time differences rather than n_H_. From this point onwards, the first sensor is considered to be the nearest to the point P_Im_ at which the impact is produced, and the first subscript of the sensor locations is avoided (P = P_i_) for the sake of clarity.

**Figure 2.**
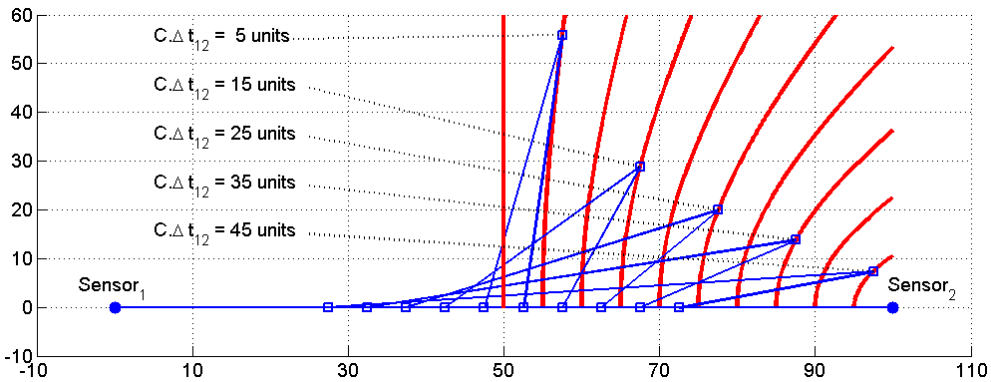
Family of hyperbolas branches for a given pair of sensors.

In accordance with the same above Equation (5), differences of time are converted into signed differences of distances that define signed hyperbola branches. Figure 2 depicts a family of signed hyperbolas when a pair of sensors (the second sensor located at P_2_ = (0 100)^T^ distance units) determines the foci of the family of the hyperbolas, while the signed distance given by Equation (4) determines the constant distance 2.a which defines each curve segment. For the sake of clarity, only positive branches whose distance differences are between 0 and 45 and are equally spaced every five distance units have been plotted.

If n sensors are located on a flat plate and all combinations are considered, the impact position is obtained with a simple computation of the intersections between n − 1 independent signed hyperbola branches. The maximum number of intersections is denoted by n_I_ and it is calculated as:

(6) $$n_{I}=\left(\begin{array}{c} n-1\\ 2 \end{array}\right)=\frac{(n-1)!}{2!\cdot (n-3)!}=\frac{\left(n-1)\cdot (n-2\right)}{2}=\frac{n^{2}-3n+2}{2}\text{for}\;n\geq 3$$

The minimum number of sensors needed for impact location on a plate is therefore n = 3, the number of independent signed hyperbola branches is n − 1 = 2, and the number of intersections is n_I_ = 1.

**Figure 3.**
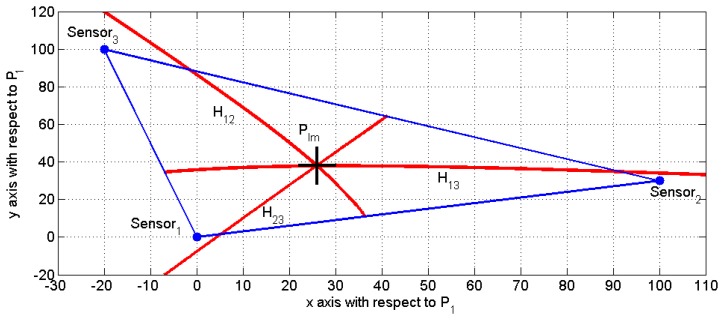
Sensor location, impact point and hyperbola intersection.

Figure 3 depicts an example of the proposed impact detection procedure. The sensors are located at: P_1_ = (0 0)^T^, P_2_ = (100 30)^T^ and P_3_ = (−20 100)^T^, and the impact position is simulated at P_Im_ = (26 38)^T^ (denoted as a large black “+”).

Three segments of signed hyperbola branches, denoted as H_ij_ for i = 1, 2, i < j ≤ 3, have also been plotted in Figure 3. Each of these segments represents the geometric locus of points with the same signed distance difference (limited by the polygon defined by the sensors). It can be clearly observed that only two of them are independent and only one intersection point is obtained, even in the case in which the speed of propagation C is not well determined.

The proof of this result is inmediate. In accordance with Equation (2):

(7) $$\Delta t_{ij}=\{\begin{array}{ccc} \Delta t_{12} & = & t_{1}-t_{2}\\ \Delta t_{13} & = & t_{1}-t_{3}\\ \Delta t_{23} & = & t_{2}-t_{3} \end{array}$$

Increment Δt_23_ can be expressed as a linear combination of increments Δt_12_ and Δt_13_ as:

(8) $$\Delta t_{23}=\Delta t_{13}-\Delta t_{12}$$

Then, only two differences of times are independent and only one hyperbola branch intersection is produced.

If four sensors are used, $n_{H}=\left(\begin{array}{c} 4\\ 2 \end{array}\right)=\frac{4.3}{2}=6$ hyperbola branches can be obtained but only n − 1 = 3 of them will be independent, and $n_{I}=\frac{3.2}{2}=3$ intersection points between pairs of independent hyperbola branches allow the position of the impact position to be obtained under the assumption that the speed of propagation C is well determined. The generalization of the previous proof when n > 3 is also immediate.

**Figure 4.**
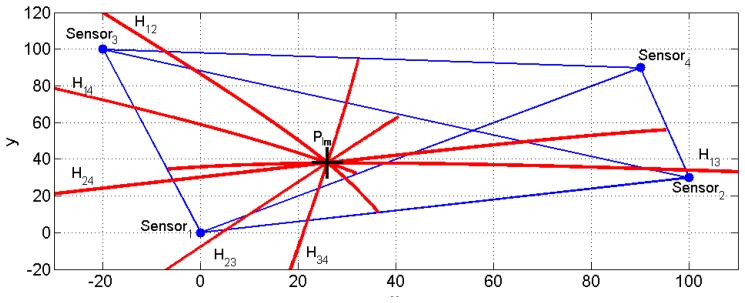
Sensor location, impact point and hyperbola intersection.

Figure 4 illustrates all of the hyperbola segments when four sensors are used. The position of the new fourth sensor is denoted as P_4_ = (90 90)^T^.

Once the impact position is known, if it is desired to calculate the absolute time instant t_Im_ when the impact occurred, this is easily computed from any of the edge signals received according to the following equation derived from Equations (1) and (2):

(9) $$t_{Im}=t_{i}-\frac{\left\|P_{\text{i Im}}\right\|}{C}\text{for}\;i=1,2,\cdots ,n$$

### 2.2. When sound Propagation Speed is not Well Known

Several research groups have noticed that when a thin plate is considered for impact detection and location, the speed of propagation is not well determined [22,23]. Under this hypothesis of a non-correct speed C with which to compute the intersection between signed hyperbola branches, the number of intersection points (denoted as n_I_ as before) increases from n_I_ = 1 to the value given in Equation (6).

When n = 4 and the speed of propagation C is not well determined either, the number of intersections increases from n = 1 to $n_{I}=\left(\begin{array}{c} 3\\ 2 \end{array}\right)=3$ in accordance with Equation (6) because all the intersections between the hyperbola branches do not coincide with a single point.

**Figure 5.**
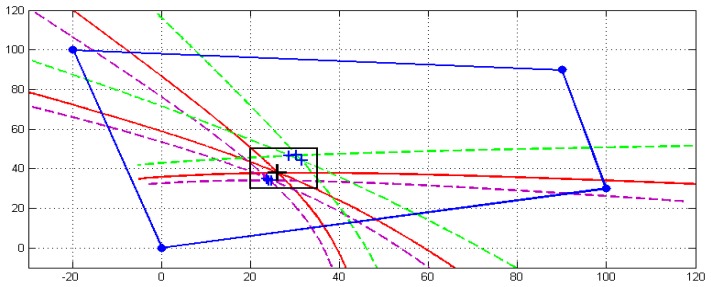
One set (correct) and two sets (not correct) of intersections with different computed speed of propagation C.

Figure 5 shows this state when n = 4. Three sets of n_I_ = 3 hyperbola branches are plotted when they are computed, assuming a perfect knowledge of the sound propagation speed C (continuous red line) and when the sound of propagation speed C is not accurately determined (dashed green and magenta lines). Each of these sets of three hyperbola branches produces n_I_ = 3 intersection points which form a triangle. These intersections are shown in the box surrounded area in Figure 5 (marked as the symbol ‘+’).

Each of the three points of each set of intersections is obtained as a function of the unknown speed of propagation c, in accordance with the following notation:

(10) $$\begin{array}{c} P_{23}(c)=H_{12}(c)\cap H_{13}(c)\\ P_{24}(c)=H_{12}(c)\cap H_{14}(c)\;\text{and}\\ P_{34}(c)=H_{13}(c)\cap H_{14}(c) \end{array}$$

**Figure 6.**
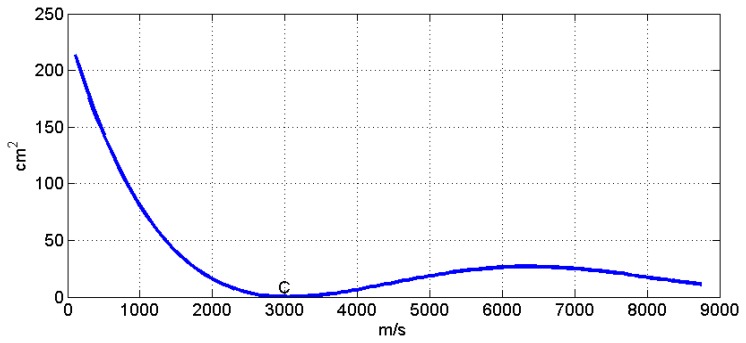
S as a function of c for 50 ≤ c ≤ 8,750 m/s in the above example. C = 3,000 m/s.

A quadratic function S which computes the sum of the squared distances among the points that determine the triangle is therefore defined as:

(11) $$S={\left[P_{23}(c)-P_{24}(c)\right]}^{2}+{\left[P_{23}(c)-P_{34}(c)\right]}^{2}+{\left[P_{24}(c)-P_{34}(c)\right]}^{2}$$

where S is a function of the unknown speed of propagation S = S(c) and measures a quadratic error of the impact point location. The value of S is of course null when the sound propagation speed c has the correct value, *i.e.*, c = C. The form of this function for different values of c can be seen in Figure 6 and has been computed from the above example in the range of 50 ≤ c ≤ 8,750 m/s by simulating an expected speed of propagation of C = 3,000 m/s.

This function has a global maximum when c ≈ 0, a global minimum when c = C, where S = 0, and different local maxima and minima values when c > C. Function S is monotone decreasing from c ≈ 0 to c = C, which provides a criterion with which to find the global minimum with any search algorithm such as the linear-dichotomy search with a computational cost of order O(N log N), the size of the searching problem being N.

If five sensors are used for an impact point detection and location on a plate, then the number of intersections increases from 1 to $n_{I}=\left(\begin{array}{c} 4\\ 2 \end{array}\right)=6$. Function C computes the squared distances between 6 intersection points, and it is possible to detect one wrong time difference and compute it by discarding this signal and obtaining the impact point position as was described above with the four remaining sensors. In conclusion, the optimum number of sensors with the proposed procedure for detecting and locating an impact on a flat plate is four.

**Figure 7.**
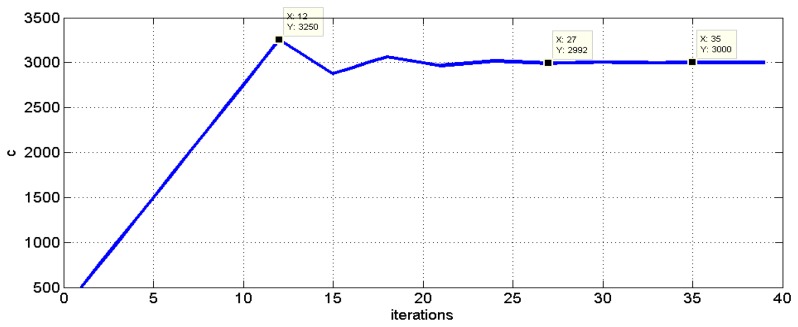
Temporal determination of C = c = 3,000 m/s for the above example.

**Figure 8.**
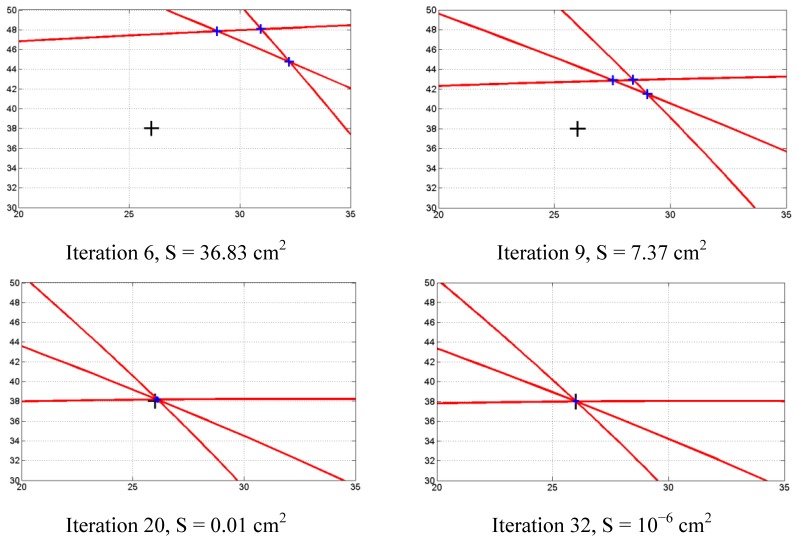
Sequence of intersection points for 6, 9, 20 and 32 iterations.

Finally, Figure 7 depicts the evolution of the speed of propagation C search algorithm as a function of the number of iterations, while Figure 8 illustrates some of the intersection points in the computation of the S function. The plotted area in Figure 8 corresponds to the black rectangle drawn in Figure 5 near the impact point.

### 3. Experimental Setup

**Figure 9.**
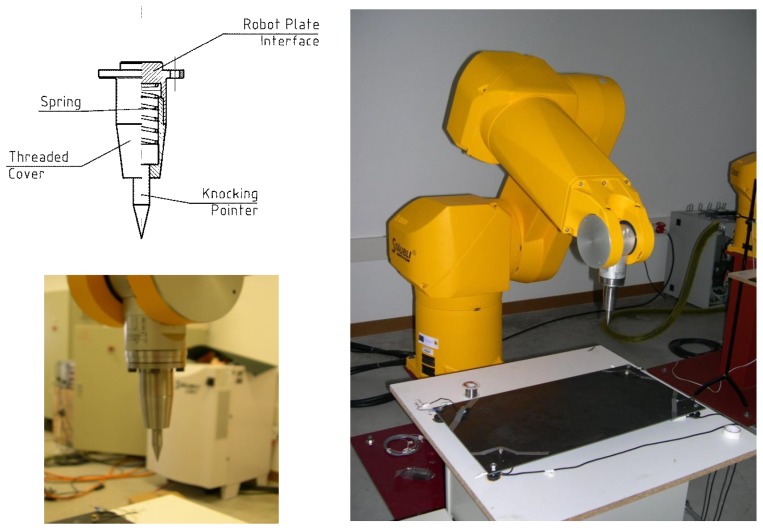
Knocking tool and its integration into the robotized setup.

The proposed theoretical procedure was validated by developing an experimental setup based on a robotized system. A Stäubli RX-130 robot and a special knocking tool added to the robot's wrist (designed and manufactured by the research group) were used to carry out the experimental validation. The knocking tool developed is shown in the top left of Figure 9. It consists of three main elements: a knocking pointer which is responsible for producing impacts, an interchangeable spring and an enclosure with a threaded cover that houses the spring and the pointer and allows coupling with the robot's wrist. The final aspect of the tool coupled with the robot's wrist is detailed in the bottom left of Figure 9. The right-hand side of Figure 9 shows the final appearance of the robot with the knocking tool when it is ready to start making impacts on the flat plate located in front of the robot.

The most interesting features of the Stäubli RX130 in our experimental validation are summarized as follows: valid workspace with six degrees of freedom, programmable motions at 13.2 m/s maximum speed of linear motion, programming facilities with which to produce precise and repetitive impacts on pseudo random pre-stored impacts points and ±0.03 mm repeatability.

A steel flat rectangular plate that was 645 mm long, 310 mm wide and 3 mm thick was supported by four mounts with acoustic isolating rubber. Four sensors were also located at the following locations (given in mm): P_1_ = (0 0)^T^, P_2_ = (450 0)^T^, P_3_ = (0 200)^T^ and P_4_ = (450 200)^T^.

**Figure 10.**
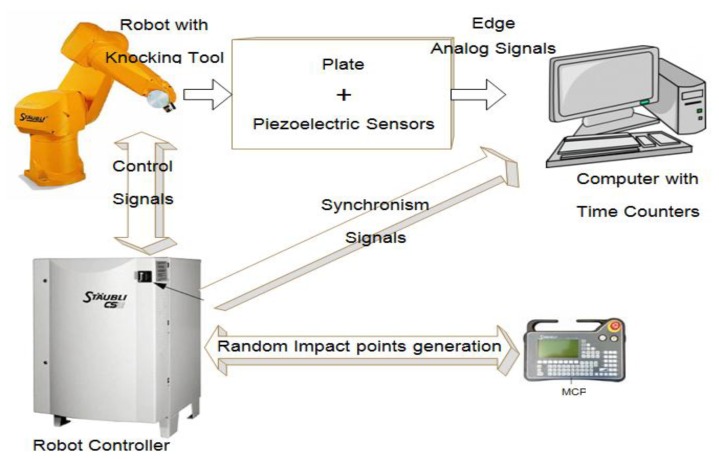
Experimental setup schematic view

A description of the experiment is illustrated in Figure 10 and related as follows: A 9 × 7 array of equally spaced points in the area bounded by the four piezoelectric sensors were automatically generated by the robot controller in a random order and with added random position increments. A start signal was produced by a user and sent from the Manual-Control-Pendant (MCP) to the controller. A synchronism signal was then sent by the controller to the computer, after which the robot produced 30 impacts (repetitive points are possible) on the plate.

The robot spent an average time of 38 seconds on any set of 30 impacts, while the signals directly measured from the piezoelectric sensors were considered to be fully damped after about 120 ms in the worst case. These experimental values of knocking and natural signal attenuation times allowed all the impacts caused on the plate to be detected and located through the application of the procedure explained in Section 2, they were subsequently compared with the random points generated that had been sent from the robot controller via serial link (RS-232) to the computer. The piezoelectric sensors were fixed and pressed against the plate (not shown in Figure 9) beforehand and then connected to the analog input of the circuit with calibrated gains, thus attaining the edge signals from the amplified voltage signals generated by the piezoelectric sensors. These edge signals were sent to the digital portion of the circuit which measured the time differences from the digital counters which were synchronized with the computer through its bus clock signal. A PC computer provided with a 32 bits PCI bus was used for this. The digital stage of the conditioner can be easily replaced with a DSP card or any FPGA based instrumentation system. Each of the analog stages of the conditioners was adjusted to detect amplified voltage signals of over 1.0 V. The average noise signal ratio measured with regard to maximum values was about −40 dB, and the absolute maximum measured noise from the piezoelectric sensors was ±30 mV, which is considered non-significant.

### 4. Experimental Results

**Figure 11.**
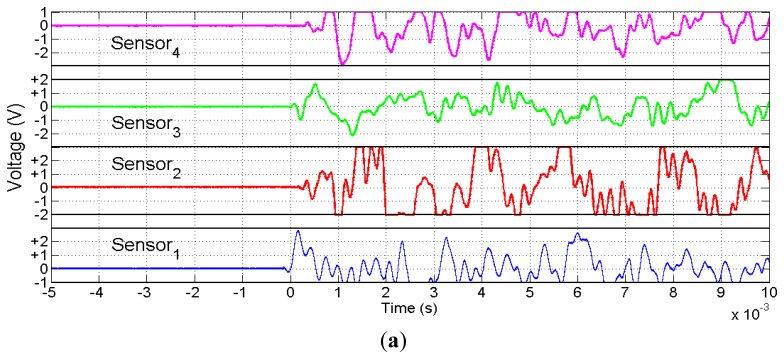

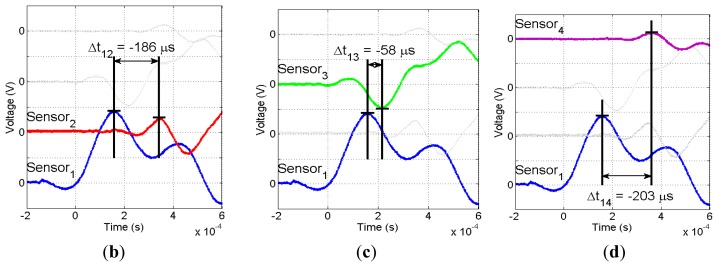
**(a)** Voltage signals from piezoelectric sensors after an impact; (**b**) Time differences at peak amplitudes of signals S_1_ and S_2_; (**c**) Time differences at peak amplitudes of signals S_1_ and S_3_; (**d**) Time differences at peak amplitudes of signals S_1_ and S_4_.

Figure 11 shows a general view of the direct voltage signals obtained from the piezoelectric sensors (Figure 11(a)) before amplification. Figure 11 (b–d) shows the same signals with an expanded time scale and two time cursors of the oscilloscope manually positioned on the maximum peak amplitude of signals between sensor pairs 1–2, 1–3 and 1–4. All this information was captured through a Tektronix DPO 4034 350 MHz oscilloscope and sent to a laptop via USB link provided with MATLAB^©^, the Instrument Control Toolbox and Tektronix VISA software. The increments of time measured for this example were Δt_12_ = −185.6 μs, Δt_13_ = −58.4 μs and Δt_14_ = −202.8 μs.

**Figure 12.**
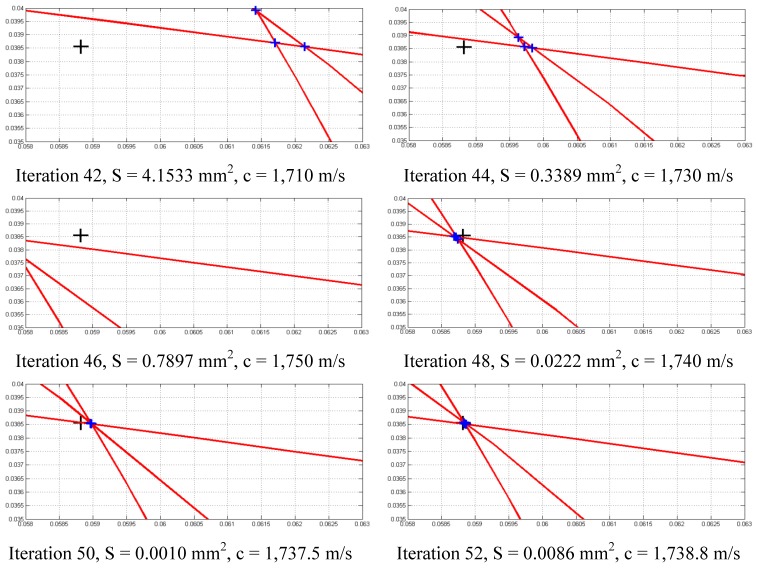

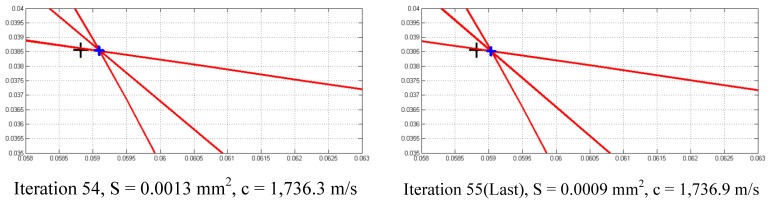
Convergence of S function around P_Im_ (distance units in m).

The impact point generated by the robot controller and then produced for the exposed example is P_Im_ = (58.82 38.56)^T^ mm with an assumed error of ±0.03 mm (robot repeatability), while the speed of sound is considered to be completely unknown. Figure 12 shows the sequence of minimizing function S for the above example. The assumed minimum speed is c_min_ = 1,300 m/s while the maximum speed is c_MAX_ = 7,000 m/s.

After 55 iterations, the algorithm is stopped because *S* is lower than a threshold value defined by the user, and the speed of propagation C is obtained, which is C = 1,737 m/s. The time differences from the impact detected provides the location of the impact detected as being (59.03 38.52)^T^ mm, and exhibits an absolute error of 0.214 mm with regard to the real impact point P_Im_ = (58.82 38.56)^T^ mm which is considered to be a good correspondence.

**Figure 13.**
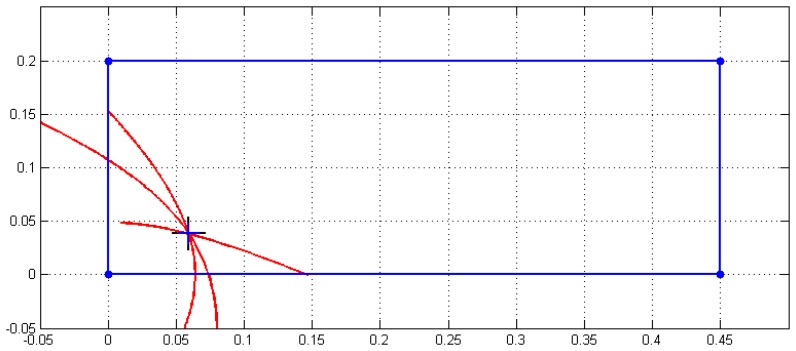
Experimental impact detection and location for the given example (distance units in m).

Figure 13 depicts the real and detected impact point (difference not visible if an enlarged scale is not used), the three hyperbola branches and the location of the four sensors. The enlarged zoom Figure 13 around P_Im_ is the same as that shown in Figure 12, when the number of iterations was 55.

**Figure 14.**
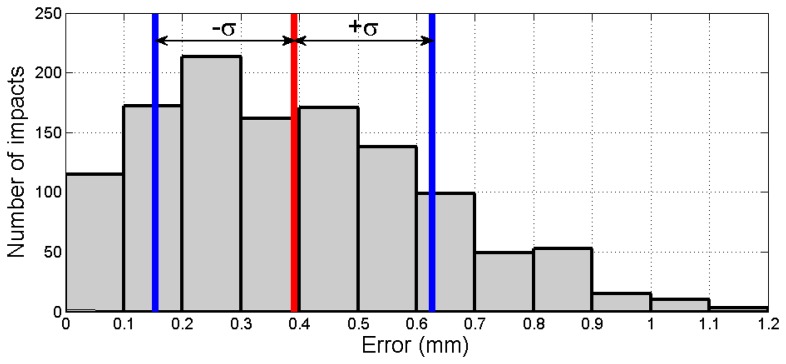
Results of the proposed methodology after 1,200 impacts.

Finally, in order to provide a better insight into the efficiency of the proposed methodology, forty tests were implemented (40 tests × 30 impacts/test = 1,200 impacts) showing the results illustrated in Figure 14. A mean absolute error of 0.391 mm with a standard deviation of σ = 0.236 mm can be obtained from this figure.

## 5. Conclusions

It is well known that if the speed of propagation of acoustical waves on a flat plate is established, then only three piezoelectric sensors are necessary to determine the position and the time instant of an impact on the flat plate. The impacts on the flat plate produce acoustic waves on the material which are detected by the piezoelectric sensors. The propagation of the acoustic waves on a plate is usually modeled as a function of the vibration modes excited by the impact, and the superposition of both, longitudinal and transversal waves, with different propagation velocities causes inaccuracies in the achievement of the true value of the speed of propagation. In these cases, it is not possible to obtain the position in and the time instant at which the impact is produced from the signals collected from only three sensors.

An iterative algorithm based on a function which quantifies the quadratic distance between the intersection points of the hyperbola branches for different values of the unknown speed of propagation allows us to obtain the accurate value of the propagation velocity of the acoustic waves and to then determine the position in and the time instant at which the impact was produced. Only four piezoelectric sensors are required to compute this function, and the optimum number of sensors is consequently considered to be four. Moreover, if the number of sensors is increased, then the effect of incorrect time measurements or sensor failures can be solved after processing the information attained from the other piezoelectric sensors.

The proposed algorithm has been validated with an experimental setup based on an industrial robot with a specially designed knocking tool and a three-stage circuit with which to convert acoustic signals into edge voltage signals let us validate the proposed algorithm. The experimental results demonstrate the good correspondence between the randomly generated impact points and the estimated impact points with the proposed estimation method.

Finally, in the future it will be necessary to explore and resolve a mathematical convergence analysis of the proposed quadratic function in order to determine that the proposed function exhibits one and only one global minimum to allow more efficient search algorithms to estimate the speed of propagation to be developed, and these are proposed as topics for our future research.
